# *rac*-Diethyl 9-hy­droxy-9-methyl-7-phenyl-1,4-diaza­spiro­[4.5]decane-6,8-dicarboxyl­ate

**DOI:** 10.1107/S1600536810054498

**Published:** 2011-01-08

**Authors:** Abel M. Maharramov, Arif I. Ismiyev, Bahruz A. Rashidov, Gunel M. Rahimova, Mirza A. Allahverdiyev

**Affiliations:** a Baku State University, Z. Khalilov St. 23, Baku, AZ-1148, Azerbaijan

## Abstract

The title mol­ecule, C_21_H_30_N_2_O_5_, is chiral with four stereogenic centres. The crystal is a racemate and consists of enanti­omeric pairs with the relative configuration *rac*-(6S*,7R*,8R*,9S*). The ethyl fragment of the eth­oxy­carbonyl group at position 6 is disordered in a 0.46 (3):0.54 (3) ratio. The crystal structure features inter­molecular N—H⋯O. Intra­molecular O—H⋯N and N—H⋯O hydrogen bonds also occur.

## Related literature

For general background to the biological activity of β-cyclo­ketoles and their nitro­genous derivatives, see: Krivenko *et al.* (2003 ▶).
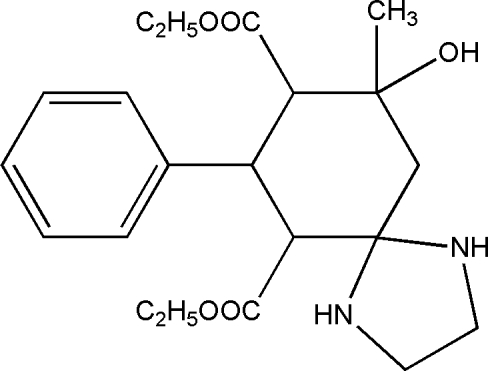


## Experimental

### 

#### Crystal data

C_21_H_30_N_2_O_5_
*M* *_r_* = 390.47
Triclinic, 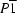

*a* = 9.4140 (17) Å
*b* = 10.7606 (19) Å
*c* = 10.7874 (19) Å
α = 103.000 (4)°
β = 97.413 (4)°
γ = 97.736 (4)°
*V* = 1040.6 (3) Å^3^
*Z* = 2
Mo *K*α radiation
μ = 0.09 mm^−1^
*T* = 296 K
0.20 × 0.20 × 0.15 mm

#### Data collection

Bruker APEXII CCD diffractometer
Absorption correction: multi-scan (*SADABS*; Bruker, 2001 ▶) *T*_min_ = 0.983, *T*_max_ = 0.987
8475 measured reflections
4252 independent reflections
2225 reflections with *I* > 2σ(*I*)
*R*_int_ = 0.065

#### Refinement

*R*[*F* ^2^ > 2σ(*F* ^2^)] = 0.068
*wR*(*F* ^2^) = 0.175
*S* = 1.00
4252 reflections
288 parameters
45 restraints
H atoms treated by a mixture of independent and constrained refinement
Δρ_max_ = 0.30 e Å^−3^
Δρ_min_ = −0.25 e Å^−3^

### 

Data collection: *APEX2* (Bruker, 2005 ▶); cell refinement: *SAINT-Plus* (Bruker, 2001 ▶); data reduction: *SAINT-Plus*; program(s) used to solve structure: *SHELXL97* (Sheldrick, 2008 ▶); program(s) used to refine structure: *SHELXS97* (Sheldrick, 2008 ▶); molecular graphics: *SHELXTL* (Sheldrick, 2008 ▶); software used to prepare material for publication: *SHELXL97*.

## Supplementary Material

Crystal structure: contains datablocks global, I. DOI: 10.1107/S1600536810054498/si2321sup1.cif

Structure factors: contains datablocks I. DOI: 10.1107/S1600536810054498/si2321Isup2.hkl

Additional supplementary materials:  crystallographic information; 3D view; checkCIF report

## Figures and Tables

**Table 1:** Hydrogen-bond geometry (Å, °)

*D*—H⋯*A*	*D*—H	H⋯*A*	*D*⋯*A*	*D*—H⋯*A*
O5—H5*O*⋯N4	0.87 (3)	1.87 (3)	2.714 (4)	163 (3)
N4—H4*N*⋯O2	0.86 (4)	2.23 (4)	2.971 (4)	144 (4)
N1—H1*N*⋯O4^i^	0.93 (3)	2.32 (3)	3.113 (3)	143

Symmetry code: (i) 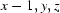.

**Fig. 1.**
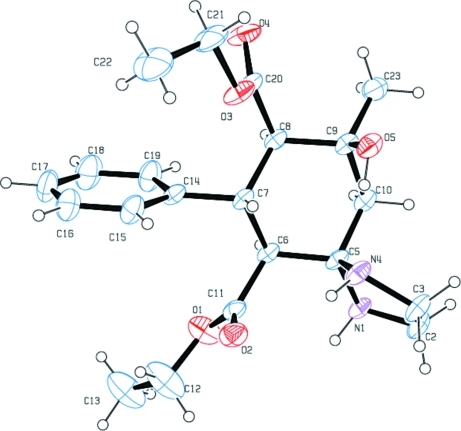
The molecular structure of the title compound, with the atomic numbering scheme. Displacement ellipsoids were drawn at the 30% probability level.

**Fig. 2.**
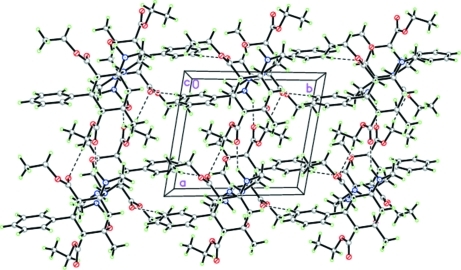
The hydrogen-bonded (dashed lines) packing in the title compound. H atoms not involved in hydrogen bonding have been omitted for clarity.

## supplementary crystallographic information

### Comment

Established that β-cycloketoles and their nitrogenous derivatives possess a wide spectrum of biological activity (Krivenko *et al.* 2003). The reactions of β-cycloketoles with ethilendiamine possibly leading to valuable compounds of practical use remain unexplored. Reaction β-cycloketoles with ethilendiamine has not been studied. Several reaction paths may be expected: one or two reactive centers of the substrate and reagent may be involved. Enamines or the products of heterocyclization or spirocyclization may be produced.

In the title compound, C_21_H_30_N_2_O_5_ (I), the cyclohexane ring adopts a chair conformation. The structure of (I) is reported here (Fig. 1). The crystal structure involves N—H···O intermolecular and O—H···N and N—H···O intramolecular hydrogen bonds. (Table 1 and Fig. 2).

The cyclohexane ring has a chair conformation. The phenyl ring is in a pseudo-equatorial position. Torsion angle between the ethoxycarbonyl group and the phenyl substituent is C14—C7—C8—C20 is 55.4 (3) ° and C11—C6—C7—C14 is -53.4 (3) °, which indicates the pseudo-axial location of hydrogen atoms at C6 C7 and C8.

THe imidazolidine ring has a envelope conformation. The fragment of a ring N1—C2—C3—N4 is almost planar - torsion angle is -6.9 (3) °.

The molecules (I) are diastereomers and possess three asymmetric centers at th C6, C7, C8 and C9 carbon atoms. The crystal of (I) is racemate and consists of enantiomeric pairs with the relative configuration of the centers of *rac*-*6S*,7R*, 8R*, 9S*.*The two [(C7(*R*),C8(*R*)] of four stereogenic centres of (I) are of the same chirality.

### Experimental

(rac)-diethyl-4-hydroxy-4-methyl-6-oxo-2-phenyl-1,3-dicarboxylate (20 mmol), ethilendiamine (20 mmol) were dissolved in 20 ml e thanol. The mixture was stirred at 345–350 K within 10 h. After cooling to a room temperature obtained white crystals. The crystals were filtered and washed with ethanol and have been then dissolved in ethanol (50 ml) and recrystallized to yield colourless block-shaped crystals of the title compound.

### Refinement

The hydrogen atoms of the NH and OH-groups (I) molecule were localized in the difference-Fourier map and included in the refinement with fixed positional and isotropic displacement parameters [*U*_iso_(H) = 1.5*U*_eq_(C) for CH_3_-group and *U*_iso_(H) = 1.2*U*_eq_(N) for amino groups]. The other hydrogen atoms were placed in calculated positions with and refined in the riding model with fixed isotropic displacement parameters [*U*_iso_(H) = 1.2*U*_eq_(C)].

### Crystal data

**Table:**

C_21_H_30_N_2_O_5_	*Z* = 2
*M**_r_* = 390.47	*F*(000) = 420
Triclinic, *P*1	*D*_x_ = 1.246 Mg m^−^^3^
Hall symbol: -P 1	Mo *K*α radiation, λ = 0.71073 Å
*a* = 9.4140 (17) Å	Cell parameters from 1499 reflections
*b* = 10.7606 (19) Å	θ = 2.4–26.3°
*c* = 10.7874 (19) Å	µ = 0.09 mm^−^^1^
α = 103.000 (4)°	*T* = 296 K
β = 97.413 (4)°	Prism, colorless
γ = 97.736 (4)°	0.20 × 0.20 × 0.15 mm
*V* = 1040.6 (3) Å^3^	

### Data collection

**Table:**

Bruker APEXII CCD diffractometer	4252 independent reflections
Radiation source: fine-focus sealed tube	2225 reflections with *I* > 2σ(*I*)
graphite	*R*_int_ = 0.065
φ and ω scans	θ_max_ = 26.5°, θ_min_ = 2.0°
Absorption correction: multi-scan (*SADABS*; Bruker, 2001)	*h* = −11→11
*T*_min_ = 0.983, *T*_max_ = 0.987	*k* = −13→12
8475 measured reflections	*l* = −13→13

### Refinement

**Table:**

Refinement on *F*^2^	Primary atom site location: structure-invariant direct methods
Least-squares matrix: full	Secondary atom site location: difference Fourier map
*R*[*F*^2^ > 2σ(*F*^2^)] = 0.068	Hydrogen site location: difference Fourier map
*wR*(*F*^2^) = 0.175	H atoms treated by a mixture of independent and constrained refinement
*S* = 1.00	*w* = 1/[σ^2^(*F*_o_^2^) + (0.070*P*)^2^] where *P* = (*F*_o_^2^ + 2*F*_c_^2^)/3
4252 reflections	(Δ/σ)_max_ = 0.001
288 parameters	Δρ_max_ = 0.30 e Å^−^^3^
45 restraints	Δρ_min_ = −0.25 e Å^−^^3^

### Special details

**Table:**

Geometry. All e.s.d.'s (except the e.s.d. in the dihedral angle between two l.s. planes) are estimated using the full covariance matrix. The cell e.s.d.'s are taken into account individually in the estimation of e.s.d.'s in distances, angles and torsion angles; correlations between e.s.d.'s in cell parameters are only used when they are defined by crystal symmetry. An approximate (isotropic) treatment of cell e.s.d.'s is used for estimating e.s.d.'s involving l.s. planes.
Refinement. Refinement of *F*^2^ against ALL reflections. The weighted *R*-factor *wR* and goodness of fit *S* are based on *F*^2^, conventional *R*-factors *R* are based on *F*, with *F* set to zero for negative *F*^2^. The threshold expression of *F*^2^ > σ(*F*^2^) is used only for calculating *R*-factors(gt) *etc*. and is not relevant to the choice of reflections for refinement. *R*-factors based on *F*^2^ are statistically about twice as large as those based on *F*, and *R*- factors based on ALL data will be even larger.

### Fractional atomic coordinates and isotropic or equivalent isotropic displacement parameters (Å^2^)

**Table:**

	*x*	*y*	*z*	*U*_iso_*/*U*_eq_	Occ. (<1)
O1	0.3602 (2)	0.2303 (2)	0.8120 (2)	0.0732 (7)	
O2	0.36432 (19)	0.2704 (2)	0.61942 (18)	0.0567 (6)	
O3	0.91777 (17)	0.3860 (2)	0.62626 (17)	0.0562 (6)	
O4	1.06405 (19)	0.4465 (3)	0.8161 (2)	0.0732 (7)	
O5	0.7805 (2)	0.6254 (2)	0.68461 (18)	0.0491 (5)	
H5O	0.688 (3)	0.594 (3)	0.660 (3)	0.058 (9)*	
N1	0.3905 (2)	0.5684 (3)	0.8533 (2)	0.0424 (6)	
H1N	0.315 (3)	0.499 (3)	0.833 (3)	0.050 (8)*	
C2	0.3457 (3)	0.6699 (3)	0.7935 (3)	0.0564 (8)	
H2A	0.3883	0.7548	0.8481	0.068*	
H2B	0.2407	0.6629	0.7811	0.068*	
C3	0.3994 (3)	0.6504 (3)	0.6627 (3)	0.0560 (8)	
H3A	0.3178	0.6241	0.5923	0.067*	
H3B	0.4564	0.7299	0.6558	0.067*	
N4	0.4897 (2)	0.5477 (3)	0.6591 (2)	0.0429 (6)	
H4N	0.441 (3)	0.474 (3)	0.615 (3)	0.066 (11)*	
C5	0.5167 (2)	0.5335 (3)	0.7942 (2)	0.0364 (7)	
C6	0.5414 (2)	0.3967 (3)	0.8005 (2)	0.0355 (6)	
H6A	0.5591	0.3945	0.8915	0.043*	
C7	0.6766 (2)	0.3630 (2)	0.7404 (2)	0.0342 (6)	
H7A	0.6592	0.3675	0.6501	0.041*	
C8	0.8119 (2)	0.4630 (3)	0.8092 (2)	0.0376 (6)	
H8A	0.8285	0.4572	0.8991	0.045*	
C9	0.7911 (2)	0.6047 (3)	0.8112 (2)	0.0411 (7)	
C10	0.6525 (2)	0.6281 (3)	0.8664 (2)	0.0406 (7)	
H10A	0.6666	0.6229	0.9556	0.049*	
H10B	0.6369	0.7152	0.8654	0.049*	
C11	0.4127 (2)	0.2941 (3)	0.7330 (3)	0.0412 (7)	
C12	0.256 (2)	0.1166 (13)	0.7402 (10)	0.112 (8)	0.46 (3)
H12A	0.3007	0.0637	0.6758	0.134*	0.46 (3)
H12B	0.1726	0.1422	0.6967	0.134*	0.46 (3)
C13	0.210 (3)	0.0405 (15)	0.8359 (14)	0.132 (8)	0.46 (3)
H13A	0.2952	0.0263	0.8869	0.198*	0.46 (3)
H13B	0.1529	−0.0413	0.7897	0.198*	0.46 (3)
H13C	0.1541	0.0886	0.8912	0.198*	0.46 (3)
C12'	0.2401 (8)	0.1223 (6)	0.7744 (17)	0.094 (4)	0.54 (3)
H12C	0.2007	0.1075	0.6837	0.112*	0.54 (3)
H12D	0.1634	0.1405	0.8243	0.112*	0.54 (3)
C13'	0.299 (2)	0.0036 (7)	0.800 (2)	0.135 (7)	0.54 (3)
H13D	0.3728	−0.0150	0.7481	0.203*	0.54 (3)
H13E	0.2214	−0.0692	0.7781	0.203*	0.54 (3)
H13F	0.3400	0.0202	0.8894	0.203*	0.54 (3)
C14	0.6973 (2)	0.2259 (3)	0.7422 (2)	0.0410 (7)	
C15	0.6688 (3)	0.1281 (3)	0.6296 (3)	0.0551 (8)	
H15A	0.6359	0.1469	0.5519	0.066*	
C16	0.6883 (4)	0.0033 (3)	0.6304 (4)	0.0686 (10)	
H16A	0.6686	−0.0605	0.5534	0.082*	
C17	0.7361 (4)	−0.0276 (4)	0.7424 (4)	0.0756 (11)	
H17A	0.7496	−0.1116	0.7422	0.091*	
C18	0.7639 (4)	0.0667 (4)	0.8552 (4)	0.0831 (12)	
H18A	0.7956	0.0461	0.9322	0.100*	
C19	0.7453 (3)	0.1934 (3)	0.8562 (3)	0.0623 (9)	
H19A	0.7651	0.2565	0.9337	0.075*	
C20	0.9451 (3)	0.4310 (3)	0.7527 (3)	0.0441 (7)	
C21	1.0367 (3)	0.3475 (3)	0.5612 (3)	0.0775 (11)	
H21A	1.1162	0.3383	0.6235	0.093*	
H21B	1.0721	0.4128	0.5184	0.093*	
C22	0.9815 (4)	0.2206 (4)	0.4639 (4)	0.1019 (15)	
H22A	1.0557	0.1982	0.4138	0.153*	
H22B	0.8969	0.2282	0.4079	0.153*	
H22C	0.9568	0.1544	0.5078	0.153*	
C23	0.9203 (3)	0.7006 (3)	0.8929 (3)	0.0595 (9)	
H23A	0.8995	0.7868	0.9026	0.089*	
H23B	1.0042	0.6927	0.8515	0.089*	
H23C	0.9391	0.6829	0.9762	0.089*	

### Atomic displacement parameters (Å^2^)

**Table:**

	*U*^11^	*U*^22^	*U*^33^	*U*^12^	*U*^13^	*U*^23^
O1	0.0710 (13)	0.0810 (19)	0.0586 (13)	−0.0124 (13)	0.0097 (11)	0.0138 (13)
O2	0.0493 (11)	0.0633 (15)	0.0474 (12)	0.0136 (10)	−0.0061 (9)	−0.0016 (10)
O3	0.0418 (10)	0.0800 (16)	0.0534 (12)	0.0329 (10)	0.0173 (9)	0.0114 (11)
O4	0.0328 (10)	0.102 (2)	0.0834 (15)	0.0261 (11)	−0.0001 (10)	0.0167 (14)
O5	0.0466 (11)	0.0612 (15)	0.0510 (11)	0.0233 (10)	0.0172 (9)	0.0243 (10)
N1	0.0373 (12)	0.0513 (16)	0.0436 (12)	0.0239 (11)	0.0130 (10)	0.0089 (12)
C2	0.0543 (16)	0.057 (2)	0.0702 (19)	0.0360 (15)	0.0194 (14)	0.0199 (16)
C3	0.0556 (16)	0.065 (2)	0.0602 (18)	0.0365 (16)	0.0065 (14)	0.0272 (17)
N4	0.0445 (12)	0.0558 (18)	0.0309 (11)	0.0248 (12)	0.0012 (10)	0.0092 (12)
C5	0.0333 (12)	0.0494 (18)	0.0289 (12)	0.0223 (12)	0.0066 (10)	0.0048 (12)
C6	0.0328 (12)	0.0468 (17)	0.0287 (12)	0.0189 (11)	0.0048 (10)	0.0060 (12)
C7	0.0347 (12)	0.0427 (17)	0.0283 (12)	0.0194 (11)	0.0038 (10)	0.0080 (11)
C8	0.0344 (12)	0.0480 (18)	0.0330 (13)	0.0178 (12)	0.0033 (10)	0.0104 (12)
C9	0.0373 (13)	0.0495 (18)	0.0394 (14)	0.0176 (12)	0.0062 (11)	0.0109 (13)
C10	0.0411 (13)	0.0472 (18)	0.0348 (13)	0.0206 (12)	0.0053 (11)	0.0057 (12)
C11	0.0345 (12)	0.0481 (19)	0.0409 (15)	0.0189 (12)	0.0089 (12)	0.0020 (14)
C12	0.108 (9)	0.134 (15)	0.063 (8)	−0.066 (9)	0.013 (6)	0.011 (7)
C13	0.190 (18)	0.095 (11)	0.097 (8)	−0.027 (11)	0.049 (9)	0.012 (8)
C12'	0.117 (8)	0.091 (9)	0.059 (6)	−0.032 (7)	0.021 (6)	0.018 (5)
C13'	0.158 (12)	0.082 (8)	0.148 (13)	−0.023 (7)	−0.012 (9)	0.036 (9)
C14	0.0328 (12)	0.0499 (18)	0.0425 (14)	0.0192 (12)	0.0078 (11)	0.0080 (13)
C15	0.0654 (18)	0.055 (2)	0.0471 (16)	0.0271 (15)	0.0097 (14)	0.0066 (15)
C16	0.082 (2)	0.052 (2)	0.072 (2)	0.0242 (18)	0.0210 (18)	0.0047 (19)
C17	0.087 (2)	0.049 (2)	0.103 (3)	0.0321 (19)	0.022 (2)	0.027 (2)
C18	0.107 (3)	0.078 (3)	0.077 (3)	0.040 (2)	0.003 (2)	0.039 (2)
C19	0.084 (2)	0.058 (2)	0.0484 (17)	0.0287 (18)	−0.0020 (15)	0.0175 (16)
C20	0.0327 (13)	0.0464 (18)	0.0576 (17)	0.0181 (12)	0.0078 (12)	0.0150 (14)
C21	0.0517 (17)	0.095 (3)	0.096 (3)	0.0381 (18)	0.0391 (17)	0.012 (2)
C22	0.078 (2)	0.111 (4)	0.109 (3)	0.032 (2)	0.045 (2)	−0.014 (3)
C23	0.0449 (15)	0.057 (2)	0.073 (2)	0.0142 (14)	0.0044 (14)	0.0068 (17)

### Geometric parameters (Å, °)

**Table:**

O1—C11	1.312 (4)	C10—H10B	0.9700
O1—C12	1.452 (3)	C12—C13	1.524 (3)
O1—C12'	1.452 (3)	C12—H12A	0.9700
O2—C11	1.207 (3)	C12—H12B	0.9700
O3—C20	1.319 (3)	C13—H13A	0.9600
O3—C21	1.454 (2)	C13—H13B	0.9600
O4—C20	1.204 (3)	C13—H13C	0.9600
O5—C9	1.426 (3)	C12'—C13'	1.525 (3)
O5—H5O	0.87 (3)	C12'—H12C	0.9700
N1—C2	1.469 (4)	C12'—H12D	0.9700
N1—C5	1.472 (3)	C13'—H13D	0.9600
N1—H1N	0.93 (3)	C13'—H13E	0.9600
C2—C3	1.539 (4)	C13'—H13F	0.9600
C2—H2A	0.9700	C14—C15	1.385 (4)
C2—H2B	0.9700	C14—C19	1.390 (4)
C3—N4	1.479 (3)	C15—C16	1.382 (5)
C3—H3A	0.9700	C15—H15A	0.9300
C3—H3B	0.9700	C16—C17	1.361 (5)
N4—C5	1.491 (3)	C16—H16A	0.9300
N4—H4N	0.86 (3)	C17—C18	1.367 (5)
C5—C10	1.521 (4)	C17—H17A	0.9300
C5—C6	1.536 (4)	C18—C19	1.395 (5)
C6—C11	1.507 (4)	C18—H18A	0.9300
C6—C7	1.550 (3)	C19—H19A	0.9300
C6—H6A	0.9800	C21—C22	1.503 (3)
C7—C14	1.518 (4)	C21—H21A	0.9700
C7—C8	1.536 (3)	C21—H21B	0.9700
C7—H7A	0.9800	C22—H22A	0.9600
C8—C20	1.511 (3)	C22—H22B	0.9600
C8—C9	1.558 (4)	C22—H22C	0.9600
C8—H8A	0.9800	C23—H23A	0.9600
C9—C23	1.515 (4)	C23—H23B	0.9600
C9—C10	1.531 (3)	C23—H23C	0.9600
C10—H10A	0.9700		
			
C11—O1—C12	110.4 (5)	O2—C11—C6	124.7 (3)
C11—O1—C12'	124.8 (8)	O1—C11—C6	111.8 (2)
C12—O1—C12'	16.3 (9)	O1—C12—C13	107.8 (3)
C20—O3—C21	117.9 (2)	O1—C12—H12A	110.2
C9—O5—H5O	97 (2)	C13—C12—H12A	110.2
C2—N1—C5	104.2 (2)	O1—C12—H12B	110.2
C2—N1—H1N	108.9 (19)	C13—C12—H12B	110.2
C5—N1—H1N	111.4 (16)	H12A—C12—H12B	108.5
N1—C2—C3	107.0 (2)	O1—C12'—C13'	107.4 (3)
N1—C2—H2A	110.3	O1—C12'—H12C	110.2
C3—C2—H2A	110.3	C13'—C12'—H12C	110.2
N1—C2—H2B	110.3	O1—C12'—H12D	110.2
C3—C2—H2B	110.3	C13'—C12'—H12D	110.2
H2A—C2—H2B	108.6	H12C—C12'—H12D	108.5
N4—C3—C2	105.8 (2)	C12'—C13'—H13D	109.5
N4—C3—H3A	110.6	C12'—C13'—H13E	109.5
C2—C3—H3A	110.6	H13D—C13'—H13E	109.5
N4—C3—H3B	110.6	C12'—C13'—H13F	109.5
C2—C3—H3B	110.6	H13D—C13'—H13F	109.5
H3A—C3—H3B	108.7	H13E—C13'—H13F	109.5
C3—N4—C5	105.42 (19)	C15—C14—C19	117.4 (3)
C3—N4—H4N	111 (2)	C15—C14—C7	121.0 (2)
C5—N4—H4N	105 (2)	C19—C14—C7	121.6 (2)
N1—C5—N4	106.77 (18)	C16—C15—C14	121.3 (3)
N1—C5—C10	109.25 (19)	C16—C15—H15A	119.3
N4—C5—C10	108.6 (2)	C14—C15—H15A	119.3
N1—C5—C6	111.7 (2)	C17—C16—C15	120.9 (3)
N4—C5—C6	112.5 (2)	C17—C16—H16A	119.6
C10—C5—C6	107.91 (19)	C15—C16—H16A	119.6
C11—C6—C5	112.78 (19)	C16—C17—C18	119.1 (3)
C11—C6—C7	108.50 (18)	C16—C17—H17A	120.4
C5—C6—C7	110.9 (2)	C18—C17—H17A	120.4
C11—C6—H6A	108.2	C17—C18—C19	120.9 (4)
C5—C6—H6A	108.2	C17—C18—H18A	119.6
C7—C6—H6A	108.2	C19—C18—H18A	119.6
C14—C7—C8	112.20 (19)	C14—C19—C18	120.4 (3)
C14—C7—C6	110.7 (2)	C14—C19—H19A	119.8
C8—C7—C6	110.38 (18)	C18—C19—H19A	119.8
C14—C7—H7A	107.8	O4—C20—O3	123.6 (2)
C8—C7—H7A	107.8	O4—C20—C8	123.6 (3)
C6—C7—H7A	107.8	O3—C20—C8	112.8 (2)
C20—C8—C7	111.2 (2)	O3—C21—C22	108.4 (2)
C20—C8—C9	111.4 (2)	O3—C21—H21A	110.0
C7—C8—C9	112.87 (18)	C22—C21—H21A	110.0
C20—C8—H8A	107.0	O3—C21—H21B	110.0
C7—C8—H8A	107.0	C22—C21—H21B	110.0
C9—C8—H8A	107.0	H21A—C21—H21B	108.4
O5—C9—C23	106.6 (2)	C21—C22—H22A	109.5
O5—C9—C10	110.33 (19)	C21—C22—H22B	109.5
C23—C9—C10	110.1 (2)	H22A—C22—H22B	109.5
O5—C9—C8	110.8 (2)	C21—C22—H22C	109.5
C23—C9—C8	110.9 (2)	H22A—C22—H22C	109.5
C10—C9—C8	108.1 (2)	H22B—C22—H22C	109.5
C5—C10—C9	114.35 (19)	C9—C23—H23A	109.5
C5—C10—H10A	108.7	C9—C23—H23B	109.5
C9—C10—H10A	108.7	H23A—C23—H23B	109.5
C5—C10—H10B	108.7	C9—C23—H23C	109.5
C9—C10—H10B	108.7	H23A—C23—H23C	109.5
H10A—C10—H10B	107.6	H23B—C23—H23C	109.5
O2—C11—O1	123.4 (3)		
			
C5—N1—C2—C3	24.6 (3)	C23—C9—C10—C5	−177.9 (2)
N1—C2—C3—N4	−6.9 (3)	C8—C9—C10—C5	−56.6 (3)
C2—C3—N4—C5	−13.4 (3)	C12—O1—C11—O2	9.9 (12)
C2—N1—C5—N4	−33.6 (3)	C12'—O1—C11—O2	1.2 (6)
C2—N1—C5—C10	83.7 (3)	C12—O1—C11—C6	−168.6 (11)
C2—N1—C5—C6	−157.0 (2)	C12'—O1—C11—C6	−177.4 (5)
C3—N4—C5—N1	29.4 (3)	C5—C6—C11—O2	61.6 (3)
C3—N4—C5—C10	−88.3 (2)	C7—C6—C11—O2	−61.6 (3)
C3—N4—C5—C6	152.3 (2)	C5—C6—C11—O1	−119.8 (2)
N1—C5—C6—C11	59.3 (3)	C7—C6—C11—O1	116.9 (2)
N4—C5—C6—C11	−60.8 (3)	C11—O1—C12—C13	175 (2)
C10—C5—C6—C11	179.39 (18)	C12'—O1—C12—C13	−32 (3)
N1—C5—C6—C7	−178.81 (18)	C11—O1—C12'—C13'	116.7 (18)
N4—C5—C6—C7	61.1 (2)	C12—O1—C12'—C13'	86 (4)
C10—C5—C6—C7	−58.7 (2)	C8—C7—C14—C15	−128.3 (2)
C11—C6—C7—C14	−53.4 (3)	C6—C7—C14—C15	107.9 (3)
C5—C6—C7—C14	−177.81 (19)	C8—C7—C14—C19	51.7 (3)
C11—C6—C7—C8	−178.3 (2)	C6—C7—C14—C19	−72.1 (3)
C5—C6—C7—C8	57.3 (3)	C19—C14—C15—C16	−0.5 (4)
C14—C7—C8—C20	55.4 (3)	C7—C14—C15—C16	179.6 (3)
C6—C7—C8—C20	179.4 (2)	C14—C15—C16—C17	0.1 (5)
C14—C7—C8—C9	−178.55 (19)	C15—C16—C17—C18	0.4 (5)
C6—C7—C8—C9	−54.5 (3)	C16—C17—C18—C19	−0.6 (6)
C20—C8—C9—O5	57.6 (3)	C15—C14—C19—C18	0.2 (5)
C7—C8—C9—O5	−68.3 (2)	C7—C14—C19—C18	−179.8 (3)
C20—C8—C9—C23	−60.6 (3)	C17—C18—C19—C14	0.3 (6)
C7—C8—C9—C23	173.5 (2)	C21—O3—C20—O4	3.1 (4)
C20—C8—C9—C10	178.6 (2)	C21—O3—C20—C8	−177.9 (2)
C7—C8—C9—C10	52.7 (3)	C7—C8—C20—O4	−142.5 (3)
N1—C5—C10—C9	−178.1 (2)	C9—C8—C20—O4	90.6 (3)
N4—C5—C10—C9	−62.0 (3)	C7—C8—C20—O3	38.5 (3)
C6—C5—C10—C9	60.2 (3)	C9—C8—C20—O3	−88.4 (3)
O5—C9—C10—C5	64.7 (3)	C20—O3—C21—C22	135.8 (3)

### Hydrogen-bond geometry (Å, °)

**Table:**

*D*—H···*A*	*D*—H	H···*A*	*D*···*A*	*D*—H···*A*
O5—H5O···N4	0.87 (3)	1.87 (3)	2.714 (4)	163 (3)
N4—H4N···O2	0.86 (4)	2.23 (4)	2.971 (4)	144 (4)
N1—H1N···O4^i^	0.93 (3)	2.32 (3)	3.113 (3)	143

Symmetry codes:  (i) *x*−1, *y*, *z*.
